# Bio-Based Polymeric Membranes: Development and Environmental Applications

**DOI:** 10.3390/membranes13070625

**Published:** 2023-06-27

**Authors:** Mónica Morales-Jiménez, Daniel A. Palacio, Manuel Palencia, Manuel F. Meléndrez, Bernabé L. Rivas

**Affiliations:** 1 Centro Interdisciplinario de Investigación para el Desarrollo Integral Regional (CIIDIR-Unidad Oaxaca), Instituto Politécnico Nacional, Calle Hornos 1003, Colonia Noche Buena, Santa Cruz Xoxocotlán 71230, Mexico; mmoralesj@ipn.mx; 2 Departamento de Polímeros, Facultad de Ciencias Químicas, Universidad de Concepción, Edmundo Larenas 129, Casilla 160-C, Concepción 4070371, Chile; 3 GI-CAT, Department of Chemistry, Faculty of Natural and Exact Science, Universidad del Valle, Cali 25360, Colombia; 4 Departamento de Ingeniería de Materiales (DIMAT), Facultad de Ingeniería, Universidad de Concepción, Edmundo Larenas 270, Casilla 160-C, Concepción 4070371, Chile; 5 Unidad de Desarrollo Tecnológico, 2634 Av. Cordillera, Parque Industrial Coronel, P.O. Box 4051, Concepción 4191996, Chile

**Keywords:** bio-based polymer, membrane, fabrication, recovery, pollutants

## Abstract

Nowadays, membrane technology is an efficient process for separating compounds with minimal structural abrasion; however, the manufacture of membranes still has several drawbacks to being profitable and competitive commercially under an environmentally friendly approach. In this sense, this review focuses on bio-based polymeric membranes as an alternative to solve the environmental concern caused by the use of polymeric materials of fossil origin. The fabrication of bio-based polymeric membranes is explained through a general description of elements such as the selection of bio-based polymers, the preparation methods, the usefulness of additives, the search for green solvents, and the characterization of the membranes. The advantages and disadvantages of bio-based polymeric membranes are discussed, and the application of bio-based membranes to recover organic and inorganic contaminants is also discussed.

## 1. Introduction

Membrane technology is a helpful alternative to separation process (i.e., microfiltration (MF), ultrafiltration (UF), nanofiltration (NF), and reverse osmosis (RO)), and to specific applications such as heavy metal remediation, seawater desalination, gas purification, pathogen removal, wastewater treatment, juice clarification, high added value compounds recovery, chemical transformations in catalytic membranes, membrane bioreactors, dialysis, drug release, and cell culture [1,2,3,4]. However, the increase of environmental issues and affections on human health derived from the preparation and use of solid polymeric materials made from fossil sources is nowadays a general concern; some of them are global warming, marine ecotoxicity, human carcinogenic and no carcinogenic toxicity, high energy demand, and land use [2]. The main critical factors involved are the emission of volatile organic compounds, wastewater with high toxicity, a large number of spent solvents, and solid, not recyclable waste [5]. Particularly, polymeric membrane fabrication suffers many challenges to be suitable and environmentally friendly. Polymer selection, solvent selection, and electricity source selection are the factors of significant environmental impact and cost [3].

To protect the environment, green alternatives have been developed under the bioeconomy concept using renewable resources. Bio-based polymeric membranes emerge as a greener alternative to counteract the environmental problems caused by synthetic materials. This membrane type is essentially made of bio-based polymers as a polymeric matrix. Bio-based polymers are a group of natural polymers produced by biomass derived from plants, animals, or marine materials; they can be obtained through microorganisms by direct production or production of monomers by using raw materials from renewable resources. This group of polymers can be biodegradable (e.g., protein, polysaccharides, etc.) o not biodegradable (e.g., bio-polyethene, etc.) [5,6]; even more, it is desirable that the material can be microbiologically degraded into CO_2_ and water, and finally, this product can be absorbed by nature. Bio-based polymers can be divided as naturally obtained biomass polymers: regenerated cellulose, cellulose acetate (CA), starch-based materials, chitin, chitosan, modified starch, extracellular biopolymer from microalgae, and protein; bioengineered polymers: polyhydroxyalkanoates (PHAs), bacterial cellulose, and poly (glutamic acid); new metabolite polymers from biomass-originated monomers: poly (lactic acid) (PLA), poly (trimethylene terephthalate) (PTT), poly (ethylene furanoate), poly(butylene succinate) (PBS), and polyamides (e.g., Nylon); common polymers that are synthesized with monomers or precursors obtained from biomass: bio-polyethylene (bio-PE), bio-poly (propylene) (bio-PP), bio-poly (ethylene terephthalate) (bio-PET) [7]. Bio-based polymers are being explored as a potential material in membrane fabrication. To this purpose, there are still many challenges to solve to be a better option than the inorganic and fossil-based polymeric membranes, some of them are the desirable pore distribution, chemical resistance, mechanical strength, and thermal stability [8]; moreover is essential into account evaluation of life cycle which includes life cycle analysis of energy, life cycle analysis of carbon dioxide emission, and life cycle analysis of cost [2].

This review aims to summarize the advances in the fabrication of bio-based polymeric membranes. The selection of the bio-based polymer, preparation methods, and the possibility of having a completely green preparation process is analyzed. The characterization of bio-based polymeric membranes is summarized, and the application on membrane separation processes is discussed, focusing on environmental applications for inorganic and organic pollutants recovery.

## 2. Membrane Fabrication in an Environmentally Friendly Way

Conventional polymeric membranes are fabricated using synthetic polymers such as polyamide (PA), polysulfone (PS), polyethersulfone (PES), polyvinylidene fluoride (PVDF), and polypropylene (PP) [1]. Synthetic polymeric-based membranes have been pioneers in the manufacture of polymeric membranes due to properties such as supporting substructure, good mechanical properties, a reasonable degree of flexibility, good chemical resistance, tolerance to a wide pH range, and resistance to high chlorine concentrations [9]. However, obtaining polymeric membranes with reproducible characteristics in an environmentally friendly way, including a greener process and adequate waste management, is nowadays a bottleneck in membrane manufacture [10]. To achieve suitability, bio-based polymeric membranes are a potential proposal mainly due to their lower environmental impact and less negative implication to society. And although it is a novel proposal, some criteria have to be considered to achieve a greener and more comprehensive manufacturing and waste management process for bio-based polymeric membranes. In this sense, many challenges are analyzed, such as bio-based polymers selection, preparation method, the use of additives, the use of green solvents, and reducing liquid and solid waste during the fabrication process [11], padding attention to the reusability potential of the membrane, cost-effective process, and on strategies to the proper waste disposal after the useful life. Ideally, for industrial scale purposes, the membrane should show a high percentage of properties such as permeability, selectivity, mechanical and thermal resistance and have a low manufacturing cost [12].

### 2.1. Bio-Based Polymer Selection

The proper selection of the bio-based polymer is critical during the manufacture of membranes because the polymeric material must show specific characteristics such as chemical resistance, thermal resistance, and flexibility, among others [13]. Table 1 details the chemical structure and molecular weight of bio-based polymers. Moreover, other specific characteristics for membrane preparation are essential to allow potential applications such as fluid-membrane interactions, effective pore size, and membrane thickness, which are characteristics that affect permeability, while characteristics such as solute transport, pore size, and separation mechanism determine selectivity and compounds rejection, without leaving aside low fabrication cost, among others [12].

#### 2.1.1. Naturally Obtained Biomass Polymers

##### Cellulose and Its Derivatives

Cellulose comprises D-anhydroglucopyranose units linked by glycosidic β-(1–4) bonds forming chains of linear structure. This homopolymer is one of the most abundant polymers in nature. It is structurally essential to plants, algae, microalgae, and fungi. Approximately cellulose represents 40% of the carbon fraction in plants. Cotton is a potential source of cellulose, as it contains more than 90 wt% [14,15]. To expand the applications of cellulose, derivatives have been produced by chemical modification of it. Cellulosic derivatives have shown characteristics such as high stiffness, low ductility, good clarity, and narrow thermal processing window; some examples are CA, cellulose acetate propionate (CAP), cellulose acetate butyrate (CAB), methylcellulose, ethyl cellulose, hydroxypropyl cellulose, among others [5].

##### Starch and Its Derivatives

Starch is a polysaccharide composed of two major macromolecular components: amylose and amylopectin polymers. Amylose is the linear component of starch. This polysaccharide is formed by D-glucose units linked by α (1–4) bonds and can reach a molecular mass 10^6^. The branched component is amylopectin, a polysaccharide composed of D-glucose units linked by α (1–4) bonds and about 5% linked by α (1–6) bonds, and it has a molecular weight ranging from 50 to 500 × 10^6^ [16]. Starch is the main reserve material in plants, and depending on plant species, variability in polysaccharide composition, granules morphology, and granular ordered structures exist. This variability impact directly on the functional properties of the starch [17]. Functional properties such as crystallinity, swelling power, and viscosity depend on the polysaccharide composition, which means the main amylose-to-amylopectin ratio. The crystallinity decreases, and a reduction in swelling ability seems with the increasing starch amylose content [17].

Moreover, to improve and extend the applications of starch, biotechnological, chemical, and physical modifications have been explored [18]. A biotechnological modification implies control of growing conditions in the plant, seed variety selection, and genetic manipulation. The physical modification includes pregelatinization, cold water swelling, heat-moisture treatment (HMT), and dry-heating treatment (DHT) of starch. Chemical transformation of the starch includes processes such as acid hydrolysis/dextrinization, oxygenation, crosslinking, and esterification [16,19].

##### Chitin and Its Derivative, Chitosan

Chitin is a biopolymer of a lineal and crystalline structure composed of N-acetyl-2-amido-2-deoxy-D-glucose units linked by β-(1–4) bonds [20]. According to molecular chains exist, α-chitin, β-chitin, and γ-chitin [21]. This polysaccharide is considered one high-added-value compound with potential properties such as biocompatibility, biodegradability, bioactivity, and low toxicity [22]. Usually, chitin is extracted from crustaceans’ shells [23], invertebrates, insect cuticles, cell walls of fungi, green algae, and yeast [21]. Versatilely some chemical, enzymatic and physical modifications have been explored on the chitin structure to improve its functional properties, such as increasing its solubility in solvents [21], cell penetration and the ability to remove pollutants as metal ions [24]. The chemical modification includes deacetylation-degradation and the introduction of new chemical groups. Enzymatic modifications comprise depolymerization, and physical modification implies the change of the surface structure of chitin. Potentially, various deacetylated chitin derivatives can be obtained under specific alkali conditions, which means concentration, temperature, and treatment time [24]. Chitosan is a chitin derivative with a degree of acetylation between 0 and 50% [21], and it is composed of N-acetyl-D-glucosamine and D-glucosamine units linked together by β-(1–4) glycosidic bonds. Different deacetylation degrees and molecular weights allow for obtaining various types of chitosan with varying surface activities [25].

##### Extracellular Polysaccharides from Microalgae

Recently, microalgae have received much attention due to their ability to produce high-added-value products [4]. Microalgae produce high and low-molecular-weight compounds excreted to the extracellular medium as a defence response to abiotic stress [26]. *Porphyridium* species produce extracellular sulfated polysaccharides [27]. *Nostoc* sp. also produces extracellular biopolymers with antifungal, emulsifying properties, and the capacity to form potential bioactive films. *Synechocystis* sp. produces an extracellular biopolymer with emulsifying potential [28]. *Chlamydomonas reinhardtii* [29], *Synechocystis aquatilis* [30], and *Nostoc calcicola* [31], among other strains, are potential producers of extracellular biopolymer. Due to the structural complexity, the elucidation structure of these biopolymers is a scientist’s challenge. Many efforts are being explored, such as the most promising strain selection [32], optimization of the culture through abiotic parameters [33], optimization of strategies to recover the extracellular biopolymer from the water body [34], development of strategy of purification and characterization of the biopolymer [35], and an ambitious option as the genetic manipulation to overexpressed the biopolymer gen [36].

#### 2.1.2. Bioengineered Polymers

Polymer microbial biosynthesis has received more attention due to advantages such as minimal environmental pollution, high purity of natural products, and profitability [37]. Therefore, engineering technologies are being developed to produce improved microbial polymers. It includes the utility of techniques to modify the microorganism’s growth rate, induce morphological modifications of the microorganism to increase cellular storage of the polymer, and in specific cases, modify the biosynthetic pathway of biopolymers [38].

##### PHAs

PHAs are a group of biopolymers produced intracellularly by bacteria through fermentation techniques (aerobic and anaerobic conditions) [39], and cyanobacteria under specific conditions of culture as pH regulation, light-dark cycles, N and P status and different carbon sources [40]. PHAs are classified as short-chain length and medium-chain length, where short-chain PHAs are composed of 3-hydroxybutyrate and/or 3-hydroxyvalerate monomers, and medium-chain PHA composed of 3-hydroxyalkanoate monomers of 6 to 14 carbon atoms [41]. Near 100 different PHAs have been identified by various microbial genera [38]. Due to properties such as biocompatibility and biodegradability, PHAs have been applied in sustainable plastics, biofuels production, mulch films for crops, agricultural nets and grow bags, among others [42]. Nowadays, PHAs have an essential role in biomedical applications such as soft and hard tissue engineering, implantable devices, and drug delivery [43].

##### Bacterial Cellulose

Bacteria of the genera *Gluconacetobacter*, *Agrobacterium*, *Achromobacter*, *Alcaligenes*, *Acetobacter*, *Aerobacter*, *Azotobacter*, *Escherichia*, *Klebsiella*, *Pseudomonas*, *Rhizobium*, *Sarcina*, *Cyanobacter*, and Green algae can synthesize bacterial cellulose, which no contain lignin, pectin, hemicelluloses, and other biogenic products, essential criteria compared with the natural sources, where the cellulose is usually accompanied of them. Different treatments are required to separate them [15,44]. The cellulose is extruded by cells as nanofibrils; the most successful producer is *Komagataeibacter xylinus*, which is a bacteria that can assimilate glucose [45] and various carbon sources, including solid and liquid agro-industrial residues [46,47]. Bacterial cellulose presents properties such as an extremely hydrophilic surface, a high water binding capacity, and biological affinity, which are significant to specific applications such as wound dressing, tissue repair, and medical implants [15].

##### Poly(glutamic acid)

Poly(glutamic acid) as a microbial biopolymer is composed of repeating units of L-glutamic acid and D-glutamic acid connected through amide linkages between α-amino and γ-carboxylic acid groups and with a high molecular weight of approximately 10^5^–8 × 10^6^ [48]. Bacteria producers are divided into L-glutamic acid-dependent and L-glutamic acid independent [37]. *Bacillus* species are the most potential bacteria to produce and secrete poly-γ-glutamic acid, usually through fermentation [49]. This biopolymer is recognized as versatile due to properties such as no toxic, highly viscous, soluble, biodegradable, and biocompatible [37]. Many efforts have been explored to enhance yield production. Some of them are strain screening and improvement, the biosynthesis of poly(γ-glutamic acid) in different hosts, optimization of the growth medium, control of the fermentation process, and product recovery optimization [48].

#### 2.1.3. New Metabolite Polymers from Biomass-Originated Monomers

**Figure 1:**
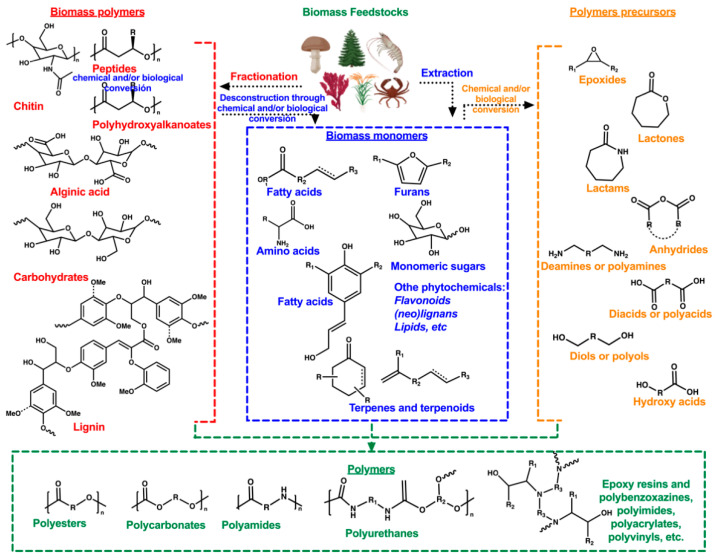
Polymers from biomass feedstocks, adapted from [50].

Nowadays, biomass feedstocks such as crops, forest waste, crustaceans, and agro-industrial residues, among others, are used to obtain building blocks for bio-based polymer production. Various methods and techniques allow to extraction or fraction of biomass into biomass monomers and biomass polymers, respectively. When these sub-products are limited, some additional treatments are done, which means that biomass polymers are fragmented through chemical or biological conversion to generate biomass monomers. These can likewise be chemically or biologically converted into polymer precursors. Finally, additional chemical transformations are required to polymerize into bio-based polymers. Figure 1 shows an illustrative manner of sourcing bio-feedstocks to produce polymers from biomass [50].

##### PLA

PLA is a linear aliphatic thermoplastic polyester. Its precursor is the acid lactic, an enantiomeric molecule, where exist L- or (S)-and D- or (R)-enantiomers [51]. The monomer can be produced biotechnologically by microbial fermentation of carbohydrates [51,52], followed by polymerization to obtain PLA. The polymerization can be by three ways such as self-condensation of individual acid lactic tapes, ring opening in lactides, or polycondensation [51]. Specific bacteria can produce acid lactic as *Lactobacillus* species.

Moreover, metabolic-engineered *Escherichia coli* has been modified to produce PLA directly. This strain can produce up to 11 wt% of PLA homopolymer using glucose as a carbon source [52]. Some efforts are still necessary for the suitable and cost-effective development of PLA polymer. In this sense, different wet waste feedstocks are explored in the production of acid lactic. About 2.1 to 3.8 Kg of dry waste is used to produce 1 Kg of PLA resin, depending on the type of feedstock [53]. Also, life cycle greenhouse gas emissions are analysed [53], and economic analysis [54]. PLA is not toxic, biocompatible, biodegradable, or compostable [51], making it a potential polymer for numerous applications in areas such as the medical industry, compost bags, and food packaging [55].

##### Bio-PTT

Bio-PTT is an aromatic polyester produced in two steps: first, lignocellulosic biomass is treated to obtain fermentable sugar and in the second step, 1,3-propanediol is produced by fermentation [56]. Secondly, PTT is melt-polymerized via a transesterification process using it and dimethyl terephthalate or via direct esterification using terephthalic acid [57]. PTT fibers have properties such as yarn bulk, crimp, stretch recovery, dyeing, stain resistance, electrostatic propensity, softness, and drape, making them attractive for applications in fiber processing, carpets, and textile fibers [57].

**Table 1:** Chemical structure and molecular weight of bio-based polymers.

Bio-Based Polymer	Chemical Structure	MW	References
Cellulose	D-anhydroglucopyranose units linked by glycosidic β-(1–4).	$1.5\times 10^{6}$ (g/mol)	[58]
Starch	Composed of amylose (D-glucose units linked by α (1–4) bonds) and amylopectin (D-glucose units linked by α (1–4) bonds and about 5% linked by α (1–6) bonds).	Amylose $10^{6}$, Amylopectin 50–500 × 10^6^ (g/mol)	[16]
Chitin	N-acetyl-2-amido-2-deoxy-D-glucose units linked by β-(1–4) bonds	HMW: >1300 kDa	[59]
Sulfated polysaccharide from *Porphyridium*	A possible acidic building unit: [(2 or 4)-β-D-Xylp-(1–3)]_m_–α-D-Glcp-(1–3)-α-D-GlcpA-(1–3)-L-Galp(1–	2.39 × 10^5^ (g/mol)	[60]
Polyhydroxyalkanoates	3-hydroxybutyrate and/or 3-hydroxyvalerate monomers.	100–450 kDa	[61]
Poly(glutamic acid)	L-glutamic acid and D-glutamic acid are connected though amide linkages between α-amino and γ-carboxylic acid groups.	10^5^–8 × 10^6^ (g/mol)	[48]
Polylactic acid	Monomer acid lactic, an enantiomeric molecule, where exists L- or (S)-, and D- or (R)-enantiomers.	183–217 kDa	[62]
Poly(trimethylene terephthalate)	Transesterification process using it and dimethyl terephthalate or via direct esterification using terephthalic acid.	117,000 g/mol	[63]
Poly(butylene succinate)	Repeating units of butylene succinate.	>65,000 g/mol	[64]
Polyamides (Nylon 6)	Repeating amide monomer units.	107,000 g/mol	[65]
Bio-polyethylene	Ethylene (C_2_H_4_) monomers.	>200,000 g/mol	[66]
Bio-polypropylene	Propylene (C_3_H_6_) monomers.	182,000–373,000 g/mol	[67]

MW: molecular weight.

##### PBS

PBS is a biodegradable aliphatic polyester composed of repeating units of butylene succinate. Usually, PBS is synthesized via co-polymerization of succinic acid or dimethyl succinate and 1,4 butanediol. These monomers can be produced from renewable and no renewable resources [68], as a green alternative succinic acid is produced by bacterial fermentation of sustainable feedstocks such as sugar-rich industrial waste [69]. Also, succinic acid is a key precursor from which bio 1,4 butanediol can be obtained by deoxidation [70]. Several bacteria and engineered bacteria can produce succinic acid or an intermediate product, e.g., cyanobacterium *Synechococcus elongates* has been metabolically engineered to produce succinate photosynthetically [71]. PBS is a promising polymer due to its thermoplastic behavior, thermo- processability, and thermos-mechanical [68].

##### Polyamides (Nylon)

Polyamides are a polymer made up of repeating amide monomer units [56]. Aliphatic polyamides are known as Nylon with an amorphous and semi-crystalline structure **[72]**, and although most polyamides are obtained by chemical synthesis, currently chemistry, modern polymer technology offers a novel approach to obtain polyamides in a biological way in which the monomers are obtained from sources of castor oil, biomass, sugars, starch or lignocellulose [56]. *Ricinus communis* plant is the main biomass feedstock for castor oil production. Under chemical transformations, it is converted into monomeric materials such as adipic acid, 1,6-hexanediamine, 1,4-butanediamine, 1,8-octanedicarboxylic acid, 1,10-decanediamine, 11-aminoundecanoic acid for the polymerization of a versatile group of polyamides [72]. Terpene-based polyamides are prepared today via polyaddition and polycondensation reactions and ring-opening polymerization. Pinenes are subjected to different reaction pathways to obtain 1,8-methane diamine (MDA), a monomer used to prepare polyamides. Also, limonene-derived diesters and diamines are used as monomers to prepare polyamides [73]. Generally, polyamides are tough, have excellent abrasion resistance, and show high tensile strength [74].

#### 2.1.4. Conventional Petrochemical Polymers from Bio-Derived Substances

##### Bio-PE

Bio-PE is a polymer with an amorphous crystalline structure and thermoplastic produced via the dehydration of bioethanol from a lignocellulosic biomass source to get bioethylene. Then the polymerization of the ethylene monomers is carried out. This process requires high pressure and temperature [56]. The polymerization treatment allows getting a variety of polyethylene polymers that can be classified according to their density and branching into high-density polyethylene (HDPE), linear low-density polyethylene (LLDPE), low-density polyethylene (LDPE), among others similar polymers of ethylene [75]. This polymer has shown excellent attributes such as mechanical properties (toughness), chemical resistance, moisture resistance, electrical insulation, and minimal coefficient of friction [76].

##### Bio-PP

Bio-PP is produced by a sequencing of steps. First, bioethanol as a green alternative precursor is obtained from the fermentation of fermentable carbohydrates. Then bioethylene is produced through dehydration of the bioethanol. A dimerization is done to get 1-butene that is isomerized to obtain 2-butene, which is carried out to a metathesis reaction to form propylene. Finally, the polymerization reaction is accomplished to produce polypropylene [56]. PP films have shown properties such as barrier, brilliance, dimensional stability, and processability, which are ideal for applications in the packaging and textile industry [76].

##### Bio-PET

Bio-PET is a thermoplastic polyester produced in a bio way. The first step is to produce the bioethanol under a careful fermentation process, and then bio-ethylene is obtained through dehydration. The bio-ethylene is converted into ethylene oxide by oxidation reaction. Through the hydration of ethylene oxide, ethylene glycol is produced. Then, the ethylene glycol is converted to terephthalic acid through polycondensation. This terephthalic acid is reacted with an alcohol (ethane-1, 2-diol) to produce monomers of PET. Finally, these are polymerized at a high 260–300 °C [56]. PET exists as an amorphous and semicrystalline material, each with ductility, stiffness, and hardness properties. Typically, this polymer is used in beverage bottles for soft drinks and water, oven trays, grill bags, audio/video tapes, and mechanical components [77]. Polyethylene terephthalate has shown a post-consumer recycling potential, and its value can be recovered by methods such as re-extrusion, mechanical, chemical (depolymerized or solvated), and energy recovery [78,79,80].

### 2.2. Characteristics, Properties and Applications of Bio-Based Polymeric Membranes

Research, application finding, and scaling of bio-based ecological membrane manufacturing processes are growing worldwide to achieve sustainability in membrane technology. Table 2 shows applications of bio-based polymeric membranes. Bio-based polymers such as cellulose and its derivatives have been well used for water desalination applications, virus removal, and wastewater treatment. Biopolymer chitin is most used for tissue engineering as a biomaterial. Chitosan and its derivatives, PHAs, and bacterial cellulose have been applied for gas separation and as a biomaterial. PLA has been used for wastewater treatment.

A novel alternative to have new properties or improve the properties of the membranes fabricated with bio-based polymers is the development of blends of bio-based polymers. The morphology of different polymers, phase adhesion, and the properties of the blend depend on the thermodynamic characteristics of different polymers. The polymer mixture based on the miscibility property is differentiated into three groups: miscible, immiscible and fractionally miscible [12]. Liu et al., 2009 prepared an antibacterial membrane blending hydrolyzed starch and chitosan. Their results showed that the mixture membrane’s elongation and water vapor transmission rate were improved [81]. Hardian et al., 2022 prepared membranes from cellulose and chitosan. Their results indicated that the membrane obtained from the mixture of cellulose with 10–25 wt% by weight of chitosan showed permeability to water from 52 to 38 L/m^2^h bar and oil removal efficiency from 73.8% to 98.6% [82].

**Table 2:** Applications of bio-based polymeric membranes.

Bio-Based Polymer	Membrane Characteristics and Properties	Applications	References
Cellulose and its derivatives	Salt rejection from 93.2% to 97.8%.	Water desalination.	[83]
	Hydrophilic, available in different pore sizes, flexibility, and associated with large filter surface area.	Virus removal capacity.	[84]
	High permeation flux, excellent separation efficiency, good flux recovery ratio. Recyclability potential and antifouling performance compared to existing commercial membranes.	Wastewater treatment.	[85]
	Water permeability of 188.0 L/m^2^h and rejection ratio of 95.2% of albumin bovine serum can be obtained with optimized conditions.	Water purification.	[86]
Chitin	Membrane with thermal stability and with good growth of NIH/3T3 fibroblast cells.	Biomaterial for tissue engineering.	[87]
	Flexible, highly porous, stable, and used as a support membrane for the growth of two mammalian cell types: NIH 3T3 and HEK293T.	Wound dressing material for tissue engineering and drug delivery.	[88]
	Membrane that can support cell attachment, proliferation, and migration also showed an excellent tensile strength of 105.7 ± 29.9 MPa.	Wound healing.	[89]
Chitosan and its derivatives	Membranes with thermal stability, and CO_2_ permeance from 15.2 to 44 GPU, and CO_2_/N_2_ selectivity from 42 to 260%.	Gas separation (CO_2_).	[90]
PHAs	Membrane of rugged structures with pores among the surface and in the cross-section. Performance of pure water permeability over 200 L/m^2^h bar and *E. coli* rejection of 99.95%.	Microfiltration, and as a biomaterial.	[91]
Bacterial cellulose	Drug-loaded membranes were flexible and with considerably higher swelling behavior.	Transdermal delivery systems for anti-inflammatory drugs.	[92]
PLA	Asymmetric membrane with albumin from bovine serum removal of 89–92%.	Wastewater treatment.	[93]
	Membranes with capacity of 52% removal rates of phosphates (PO_4_^−3^-P), and until 95.9 ± 3.1% of ammonium nitrogen (NH_4_^+^-N).		[93]

To date, the menu of applications for bio-based polymeric membranes is limited. Not all bio-based polymers have been used as polymeric matrices in the preparation of membranes, indicating an open field for research.

Comparing bio-based polymeric membranes with conventional synthetic polymeric membranes, several challenges still have to be solved to displace the manufacture of synthetic polymeric membranes because membranes made of conventional polymers derived from fossil materials offer very tempting attributes for industrial separation applications such as low manufacturing cost, excellent thermal and chemical stability, good mechanical resistance and high versatility [11].

Membrane technology, especially pressure-driven separation operation, requires a membrane with characteristics such as resistance to transmembrane pressure from 1 to 100 bar, temperature resistance, and the ability to recycle the membrane through periodic cleaning [1,4]. Membrane performance is measured as flux water and selectivity. Polyamide membranes have been excellent for large-scale desalination water with >99% salt rejection in seawater by reverse osmosis (RO) [94]. Polysulfone functionalized membranes are characterized by catalytic, ion exchange, and biomedical applications [95]. Polyvinylidene fluoride hollow fiber membranes have high hydrophobicity, a useful property for the removal of dissolved volatile species from water [96].

### 2.3. Preparation Methods of Bio-Based Polymeric Membranes

**Figure 2:**
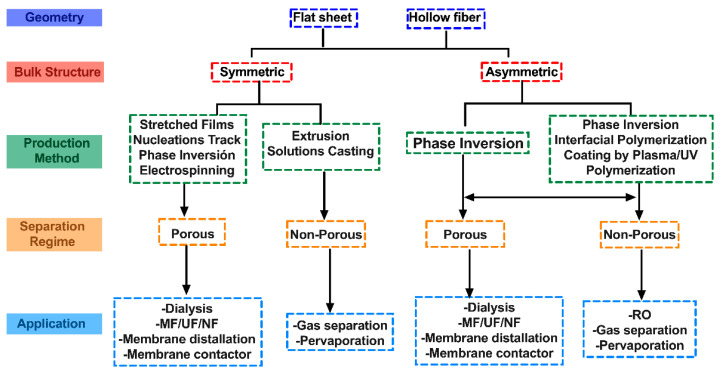
Classification of membrane fabrication, adapted from [12].

Several types of membranes are processed according to their form. It could be a plate, tube, film, or hollow fiber, combining its arrangement with membrane structures such as symmetric and asymmetric, under a separation regimen of nonporous, porous, and with modifications of charge and non-charge through common preparation methods such as a solution-casting method that involves thermally induced phase separation, diffusional induced phase inversion, composite method, casting reaction method, polyion complex method, freeze dry method, template synthesis, self-assembly, electrospinning, sintering, stretching, track-etching, and solution coatings [97,98,99]. Figure 2 shows a general classification of membrane fabrication [12].

The membrane preparation method is crucial for the membrane performance, morphological characteristics, and mechanical and thermal properties. According to Table 3, phase inversion and electrospinning are the most common methods used for bio-based membrane preparation. The characteristics depend on each bio-based polymer matrix because of its chemical structure and surface morphology. Also, novel proposals for membrane repair methods have been reported. Li et al., 2021 propose a self-crosslinking cellulose membrane prepared by blending long and short cellulose micro/nanofibers. This membrane showed that the tensile stress increases with the increase in thickness. When the thickness was 60 µm, the tensile stress was 40.8 MPa [100].

A similar panorama occurs with the preparation methods of synthetic polymeric membranes, Table 4. Even more, synthetic polymeric membrane preparation offers the possibility to make versatile modifications. PVDF membranes are commonly modified in two ways, a modification of the matrix material before membrane preparation and a modification of the surface of the product membranes [101]. Quaternary ammonium groups have been used to immobilize it on polyamide membranes surface. The modified membrane showed surface charge, hydrophilicity, and roughness [102]. Fumed silica and 1H, 1H, 2H, 2H-perfluorooctyl trichlorosilane (PFTS) were used to modify the surface of polyimide nanofibers membranes; the membranes modified showed excellent mechanical roughness, ultra-high oil-water selectivity and separation performance [103]. Optimization of the process always is necessary for the best performance according to the application.

**Table 3:** Preparation methods of bio-based polymeric membranes.

Bio-Based Polymer	Preparation Method	Type of Membrane	Observations	References
CAB	Thermally-induced phase separation (TIPS).	Hollow fiber	The roughness of thermally induced phase separation membranes was superior to the nonsolvent-induced phase separation membranes.	[104]
	Nonsolvent-induced phase separation (NIPS).			
Cellulose	Electrospinning	Thin film	The parameters optimized were a voltage of 35 kV, a tip-to-collector distance of 15 cm, and a spinning rate of 1.0 mL/h.	[105]
PLA	Phase separation	Hollow fiber	Microporous structure, high water permeability (324 ± 46 L/m^2^ h atm) and good separation performance as an ultrafiltration membrane (80% BSA rejection).	[106]
	Evaporation-induced phase separation (EIPS)	Symmetric dense flat	Membranes possess a T_g_ of around 65 °C. Thickness > 25 µm showed high CO_2_/CH_4_ ideal selectivity (220–230) and CO_2_ permeability ~11 Barrer.	[107]
Lignocellulosic acetylated	Evaporation-precipitation	Thin film	Nano-porous membrane with 169.27 nm of total roughness. Good operational performance of nanofiltration (98.47% of fluoride rejection).	[108]
Bio-polyamide 56 (PA56)	Electrospinning	Nanofiber	PA56 attached with alginate and poly-(hexamethylene biguanide) showed antibacterial activity against *Escherichia coli* (97%) and *Pseudomonas putida* (100%).	[109]
Poly(hydroxybutyrate-co-hydroxyvalerate)	Phase inversion	Asymmetric	The membrane is permeable to water up to 350 L m^−2^h^−1^bar with a pore size in the range of UF/MF.	[110]
	Evaporation-induced phase separation	Porous	Rugged structure with pores among the surface and in the cross-section. 18.0 ± 0.6 µm of membrane thickness, 9.0 ± 0.5% of porosity, 4.2 ± 2.6 L m^−2^h^−1^bar^−1^ of water permeability, and 95.0 ±2.80% of *E. coli* rejection.	[91]
Acetylated cellulose ether	Solvent evaporation	Dense thin-film	Water uptake of around 11–12 wt%. The pore diameter of 0.58–0.62 nm. Low water permeability (~10^−7^ cm^2^/sec).	[111]
	Phase inversion	Microporous asymmetric	Membrane characteristics such as a thicker, dense top layer, hindered macrovoid formation, and lower porosities depend on polymer concentrations.	
Extracellular biopolymer from microalgae	Solvent evaporation	Thin film	Transparent and flexible biofilms with pores and cracks.	[28]
PBS	Electrospinning and oxygen plasma treatment	Nanofibrous	Super hydrophilic membranes.	[112]

**Table 4:** Preparation methods of synthetic polymeric membranes.

Synthetic Polymer	Preparation Method	Type of Membrane	Observations	References
Poly(vinylidene fluoride) (PVDF)	Melt-spinning and stretching process	Hollow fiber	The membranes exhibited excellent tensile strength in the 23.0 to 62.6 MPa range. Membranes prepared with stretching 100% were about 0.317 µm, which showed a high dye rejection (<93.9%) for direct black 19.	[113]
Polyamide-PVDF	High-temperature rapid non-solvent-induced phase separation	Hollow fiber with bicontinuous structure	The results showed that the stock solution must not have a gelation temperature, and the membrane produced at an outer coagulation solution temperature higher than the upper critical solution temperature of the stock solution.	[114]
Polyimide	Electrospinning combined with surface modification	Fibrous membrane	Membranes were modified with fumed silica and 1H, 1H, 2H, and 2H-perfluorooctyl trichlorosilane (PFTS). The membranes showed ultra-high oil-water separation performance. The flux of heavy oil can reach 271.36 L/m^2^h under 25 KPa.	[103]
Polyestirene integrated with natural zeolite particles	Electrospinning	Fiber	The membrane showed a smooth surface with microdomains. The product integrated with 30 wt% zeolites had the best performance in the desalination of artificial seawater, 82.63% decrease in conductivity.	[115]
Nanocomposite polyamide 6 with intercalated silicate sheets	Thermally-induced phase separation (TIPS)	Hollow fiber	The membrane, whose composition was 50% nanocomposite, exhibited methanol permeance of 0.1 L/m^2^ h bar and vitamin B_12_ rejection of over 99.0%.	[116]
Polysulfone	Phase inversion process	Asymmetric porous	The membranes showed pure water flux of 118.5 to 695.65 L/m^2^ h at 240 kPa, porosity of 0.38 to 0.61, and bovine serum albumin rejection of 40% to 64.52% at pH 9.3.	[117]
Polysulfone/Cellulose nanofibers	Phase inversion		The membranes reinforced with cellulose nanofibers at less than 0.5wt% was the best homogeneous dispersed. This prepared membrane showed 3.2 nm of average pore size.	[118]
Polysulfone	Non-solvent coagulation bath-induced phase inversion		The optimum membrane was founded when the immersion was at 1% Na_2_SO_4_. This membrane achieved a high permeation of water flux, it was 208.75 L/m^2^ h, and the highest rejection of humic acid, it was 99.54%.	[119]
Polyethersulfone	Non-solvent-induced phase separation	Hollow fiber	It was observed that the addition of o-xylene as an additive to the cast solution reduces water permeability and membrane pore size and increases membrane strength and water contact angle.	[120]

**Figure 3:**
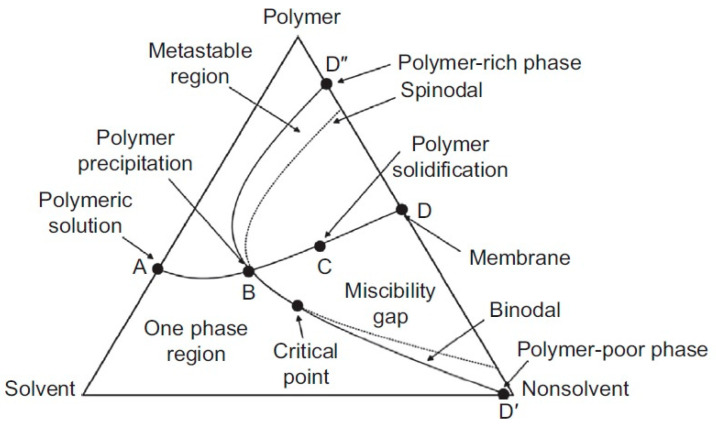
Ternary phase diagram for membrane creation by phase inversion [13].

Briefly, *phase inversion* is based on the principle of precipitation by immersion, where the polymer solution becomes thermodynamically unstable and tends to phase separation. This continuous polymer-rich phase is surrounded by a polymer-poor interface. There are four techniques to drive phase inversion of polymer solutions to create membranes such as thermally induced phase separation (temperature), diffusional induced phase separation (nonsolvent), dry casting, drying induced phase separation (evaporation), and vapor induced phase separation (nonsolvent vapor) [99]. A ternary phase diagram (Figure 3) is used to describe and understand the membrane formation when the three components (polymer, solvent, and nonsolvent) are involved. The regions inside the triangle indicate the mixture of the components. Three regions can be visualized, one phase where the three components are soluble, the metastable region, and the two-phase region where the solution begins to separate into two phases, one solid (rich in the polymer) and the other liquid (low in the polymer). The precipitation process and membrane creation occur along the curve line A to D [13].

Membranes with good mechanical stability are prepared by thermally induced phase separation, where a polymer solution is brought to high temperature using a high-boiling solvent. A membrane prepared by nonsolvent-induced phase separation is the immersion of a polymer solution in a coagulation bath containing a non-solvent. After immersion, the polymer solution becomes metastable, and demixing takes place. Two types of demixing can occur, instantaneous and delayed, and depending on the type of demixing, it can lead to two types of membrane. Instantaneous demixing produces membranes with a fine porous top layer and a finger-like macro void structure, and delayed demixing produces membranes with a dense skin layer and sponge-like substructure. A membrane prepared by evaporative-induced phase separation is accomplished by the evaporation of a solvent from a polymer solution, resulting in the precipitation of the polymer, and dense anisotropic membranes are generally obtained. The vapor-induced phase separation allows for obtaining porous membranes. For this process, the polymer solution is placed in a steam system produced by a non-solvent, generally water [13].

**Figure 4:**
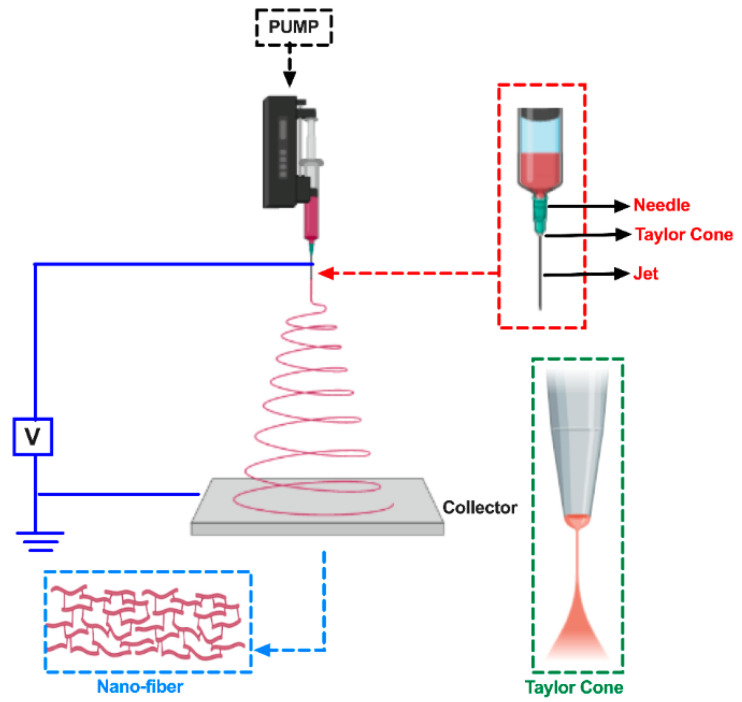
Electrospinning process, adapted from [122].

*Electrospinning,* on his part, is a relatively new technique and is widely used to fabricate non-woven fibrous membranes with tunable fibers diameter from 10 nm to 10 µm [121]. Due to the versatility of this technique, it is possible to obtain porous, permeable membranes with a surface area of 100 nm–1000 m^2^/g and with the possibility of adjusting the pore size and thickness of the membrane [122] (Figure 4). Operationally, the polymer solution is dispersed through an electrostatically charged needle, and a Taylor cone is formed through the increased voltage applied to the charged needle, finally depositing the fibers in a counter-charged collector [121].

The optimization of the electrospinning process is based on the control of parameters such as solution properties (viscosity, conductivity, dielectric constant, and surface tension), process conditions (voltage, flow rate, needle diameter), and chamber weather conditions [122]. Zhang et al., 2018 made a cellulose nanofiber membrane via the electrospinning method. They optimized the parameters and used tetra butyl ammonium chloride (TBAC) as a king of organic branching salt, it was dissolved in the cellulose solution, and the electrical conductivity of the solution was incremented. The optimal morphological structure of the membranes was founded at a voltage of 35 kV at a concentration of 0.1 mol/L of TBAC, 15 cm distance from the tip of the capillary to the collector, and a rotation rate of 1.0 mL/h [105].

### 2.4. The Utility of the Additives in the Preparation of Bio-Based Polymeric Membranes

Nowadays, additives are a good alternative to improve or expand the properties of the membranes for new applications. The additives are designed mainly to solve challenges such as forming pores, increasing permeability, hydrophilicity, and antifouling and antibacterial properties. Some novel polymeric additives for this purpose are poly (vinylpyrrolidones) (PVP), poly (ethylene glycol) (PEG), chitosan, polyamide, poly (ethylene oxide) (PEO), poly (vinyl alcohol) (PVA), poly (acrylic acid) (PAA), poly (acrylamide) (PAM), N-(2-hydroxypropyls) methyl acrylamide (HPMA) [12]. Regarding the preparation of bio-based membranes, Tomietto et al., 2020 studied the effect of adding additives such as polyvinylpyrrolidones and polyethylene glycols in the membrane preparation of poly (hydroxybutyrate-co-hydroxy valerate). They found that both additives can increase membrane porosity [91]. Shen et al., 2019, synthesized a new polymer additive via one-step esterification between cellulose acetate and perfluoroalkyl polyethoxy acetic acid. This new material has both hydrophilic and oleophobic groups. The membranes showed good antifouling properties and excellent anti-oil performance that 92.5% of oil rejections [123]. Wang et al., 2019 made a hybrid membrane of cellulose acetate and MIL-53(Fe) to improve the selectivity and permeability performance of the forward osmosis membrane. They showed a hybrid membrane of 78.6 ± 1.8% porosity and 115.7 ± 3.2 µm thickness [124].

### 2.5. Green Solvents as a Sustainable Approach in the Preparation of Bio-Based Polymeric Membranes

So far still, there are many challenges to solve to reach an environmentally friendly way and staff security during the membrane preparation, one of them being toxic solvents [11]. Although a process without solvents will be ideal, always is not possible. Green solvents such as water, bio-sourced solvents (e.g., Cyrene^TM^, isosorbide, methyl lactate, γ-valerolactone, N,N-dimethyl lactamide, succindiamide, glycerol derivatives, 2-methyl tetrahydrofuran (2-MeTHF)), nontoxic synthetic organic solvents (dimethyl sulfoxide), nonionic synthetic organic solvent (Rhodiasolv^®^ PolarClean), deep eutectic solvents, and ionic liquids arise an alternative to substitute classical toxic ones [125]. Kachhadiya and Murthy 2023 developed a novel strategy to add deep eutectic solvents (chlorine chloride and ethylene glycol) into the chitosan polymer matrix, and they found that the incorporation of deep eutectic solvents and the additive MIL-53(Fe) enhances the separation properties of the membranes obtained [126]. Tomietto et al., 2022 prepared membranes using poly (hydroxybutyrate-co-hydroxyvalerate) as a polymer and Cyrene^TM^ as a solvent. Their results showed that the membranes obtained were porous to dense with applicability in the pervaporation process [110]. Papchenco et al., 2022 studied the use of dimethyl carbonate to prepare PHA films, which showed transport properties similar to those obtained with the more toxic CHCl_3_ [127]. Methyl lactate was used as a green solvent, and 2-methyltetrahydrofuran was used as a green co-solvent by Rasool et al., 2020 to prepare cellulose acetate membranes with applicability in the nanofiltration process [128]. Although to date is complicated to get a membrane fabrication completely bio-based, Rasool and Vankelecom, 2021 showed the preparation of nanofiltration membranes under a full bio-based concept, using cellulose acetate as polymer, glycerol derivatives (monoacetin, diacetin, and triacetin) as solvents, and 2-methyltetrahydrofuran (2-MeTHF) as co-solvent, also in a via non-solvent induced phase separation. They found that the best membrane was obtained using diacetin as a solvent and 2-MeTHF as a co-solvent with permeation ranging from 5.5 to 12.8 L/m^2^h bar for membrane with >90% of rejection of dye Rose Bengal from aqueous solution [129].

### 2.6. Characterization of Bio-Based Polymeric Membranes

An integral membrane characterization implies knowing the morphological characteristics of the membrane, crystal structure, functional groups, chemical composition, and the performance characteristics of the membrane process during the separation of molecules [98,130]. The crystal structure is classically studied through X-ray diffraction (XRD); for determined functional groups, techniques such as Fourier-transform infrared (FTIR) spectroscopy, Raman spectroscopy, and nuclear magnetic resonance spectroscopy (NMR) are used. Also, techniques such as energy-dispersion X-ray (EDS), X-ray fluorescent (XRF), and X-ray photoelectron spectroscopy (XPS) are useful for measuring chemical composition [130].

Particularly, morphological parameters such as gravimetric porosity, pore size, pore distribution, tortuosity, surface wettability, roughness, surface tension, surface charge, surface functional group, molecular weight cut-off, and thickness are characterized through several techniques such as scanning electron microscopy (SEM), atomic force microscopy (AFM), confocal scanning laser microscopy (CSLM), X-ray computed micro-tomography (micro-CT), nuclear magnetic resonance (NMR), spin-echo small-angle neutron scattering (SESANS) and magnetic small-angle neutron scattering (MSANS) [98].

**Figure 5:**
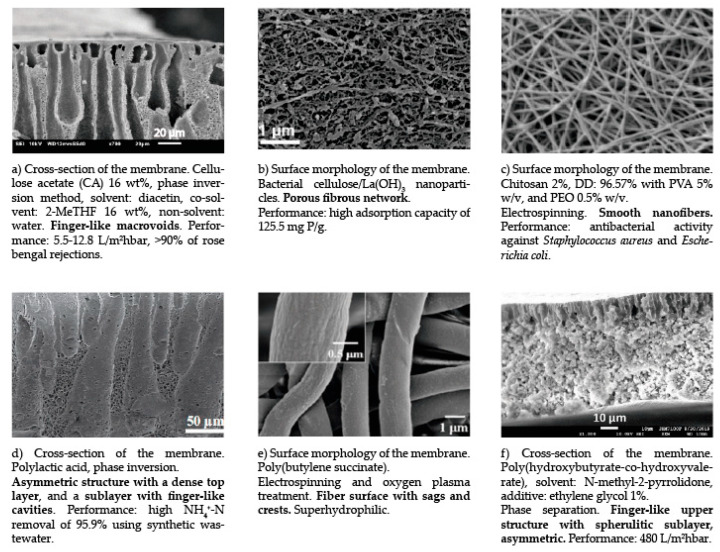
Image SEM of bio-based polymeric membranes, (**a**) [129], (**b**) [131], (**c**) [132], (**d**) [133], (**e**) [112], (**f**) [134].

SEM images help study dense surfaces, pore size measurements, and membrane thickness. A porous membrane is characterized by having a uniform structure of small holes [130]. SEM images of the cross-section and surface morphology of the bio-based polymeric membranes are shown in Figure 5.

Characterization of the membrane microstructure (such as pore size distribution and pore connectivity between pore and surface reactivity) is essential for specific membrane performance and parallel for specific applications [135]. Several pore-forming agents have been used to manipulate the pore fabrication of the membrane, which can be used through three methods: adding a pore former during the polymerization, using thermally unstable components in a polymer blend, and using selective decomposition of thermally unstable block copolymer [12]. The porous materials such as catalysts, adsorbents, oxides, carbons, zeolites, organic polymers, and soils are included in the membrane preparation based on the properties conferred to the membrane such as high surface area, relatively low stiffness, shape selectivity, and permeability [135].

**Figure 6:**
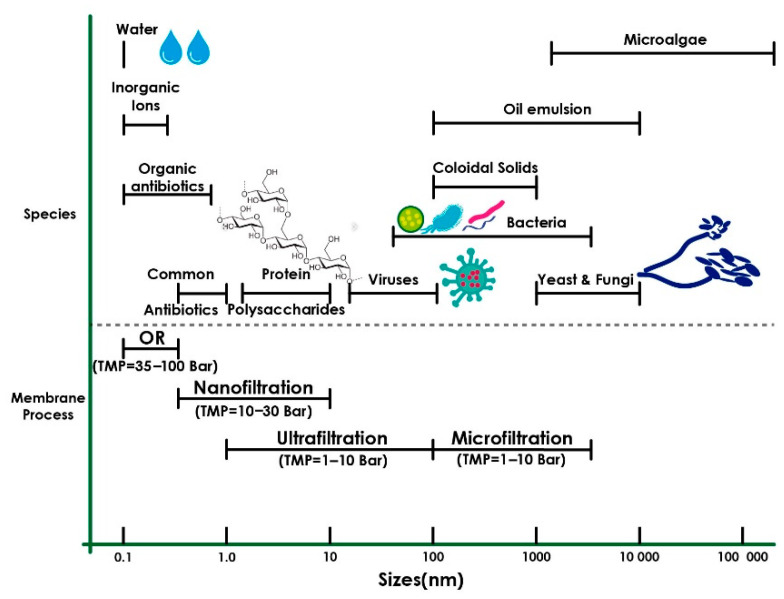
Classification of membrane process according to the pore size [4].

The pore size is used to classify the membranes when the application is separation technology. The classification for pressure-driven processes is MF, UF, NF, and RO, which are useful for the recovery of compounds. Figure 6 shows the classification of the membrane process according to the pore size [4].

On the other hand, the porous characteristics are directly related to the mechanical and thermal properties of the membrane [135]. Depending on the membrane preparation method, pore modifications occur. Lin et al., 2023 showed that during the phase inversion, the evaporation of the solvent induces a decrease in the pore size in the cellulose acetate membrane. The pore changes from an asymmetrical structure to a sponge-like pore structure. Hence the importance of the solvent; also, this fact determines the mechanical properties of the final membrane, and the researchers found that the combination of two solvents improved the tensile strength a 48% [86]. Tan et al., 2022 prepared a porous fibrous membrane of bacterial cellulose/La(OH)_3_ for phosphate removal.

Moreover, they evaluated the stability of the membrane in water through the mechanical properties in dry and wet conditions. They showed a tensile strength of 19.1 MPa and an elongation at break of 3.3% at 50% RH, and when the sample was wetted with water, the tensile strength was 12.1 MPa, and the elongation at break was 6.8%. This study shows evidence of the resistance of the membrane to wet conditions [131].

To improve the mechanical properties of the membranes, Zhou et al., 2021 prepared a cellulose acetate membrane and evaluated the effect of glycerol as a plasticizer on the mechanical properties. They found that the tensile stress in the membrane with glycerol 5% was 26.2 MPa, and without glycerol was 9.4 MPa [136].

The membrane characterization process can be evaluated through its productivity and selectivity [98], also through the analysis of parameters such as selection of feed flow, water permeability, fouling index, cleaning efficiency, and feed properties (pH, temperature, diffusivity, ion concentration, and surface content) [137].

## 3. Environmental Applications of Bio-Based Polymeric Membranes

Nowadays, membrane technology is a useful and highly efficient tool for the recovery of compounds from water bodies through the filtration and adsorption processes, also gas purification; focusing on environmental remediation and sustainability, these compounds include a variety of contaminants such as heavy metals (e.g., arsenic, copper, mercury, lead, nickel, and chromium), dye materials (e.g., azo dyes), biochemical compounds (e.g., peptides, enzymes, amino acids, proteins, polysaccharides, hormones, nucleic acids, and lipids), pharmaceutical materials (e.g., antibiotics, acetylsalicylic acid, paracetamol, and ibuprofen), nutrients from wastewater (e.g., carbon, nitrogen, and phosphorous compounds), drinking water contaminants (e.g., ammonia), air pollutants, among others [138,139].

Bioremediation is, to date, a no invasive and environmentally friendly manner to repair contaminated groundwater or soil [140]. Table 5 shows the use of bio-based membranes to remove contaminants from water through the recovery/adoption process. The bioremediation application of bio-based membranes is still limited and in progress. The bio-based polymer must be modified or supplemented with additives to improve its molecular interaction properties and retention of organic contaminants, such as dyes and bovine serum albumin, and inorganic contaminants, such as heavy metals. Chitin and its derivative, chitosan are the bio-based polymer most explored, however, exists more bio-based polymer are to be explored. Also, the operational parameters of the separation process need to be optimized.

## 4. Membrane Fouling as a Bottleneck on Membrane Technology

During a pressure-driven separation process, membrane fouling is inevitable, for which events such as complete pore clogging by particles, internal pore clogging, partial pore clogging, adsorption, chemical interaction, and cake formation on the membrane surface occur. The direct consequence of membrane fouling is decreased membrane performance (permeability and selectivity) and membrane life [1], impacting economic and environmental items. Three types of fouling are recognized: inorganic fouling, organic fouling, and biological fouling. The parameters that have an influence on the membrane fouling are membrane properties (e.g., hydrophobicity, roughness, charge, pore size, surface energy), foulant characteristics (concentration, solubility, charge, size), feed characteristics (viscosity, ionic strength, foulant kind, pH), and operation conditions (temperature, pressure, flow velocity) [146,147]. Some strategies have been developed to control the fouling index, e.g., hydrodynamic management, back flushing and pulsing, membrane surface modification, feed pretreatment, flux control, and effective membrane cleaning [1].

For high salinity wastewater treatment, preparing anti-fouling membranes is nowadays a promising purpose. To this, minimizing the interaction between membrane and foulant is analyzed, where a more hydrophobic membrane is commonly desirable [146]. Guo et al., 2023 prepared a superhydrophobic hollow fiber membrane based on polypropylene [148]. Under this concept of development of anti-fouling membranes, Ghiggi et al., 2017 prepared a modified membrane of polyethersulfone with N-phthaloyl-chitosan, which showed the anti-fouling property and performance of pure water permeability of 134.5 to 167.7 L/m^2^h [149]. Hu et al., 2021 developed a bacterial cellulose/polydopamine/reduced graphene oxide composite membrane. The membrane exhibited high permeability, 1149.3 L/m^2^h under 0.1 MPa [150]. The search for alternatives to counteract membrane fouling is still a constant challenge in membrane technology.

## 5. Conclusions and Future Perspectives

Membrane technology is today a potential strategy for the recovery of compounds due to its selectivity, cost-effectiveness, and environmentally friendly. Several bottlenecks must be solved to be sustainable. One of them is membrane fabrication. Over the course of this review, bio-based polymeric membranes were documented, along with their advances and applications, particularly environmental ones. Nowadays, the investigation about it is growing, considering challenges such as bio-based polymer selection, characteristics, properties, preparation methods, characterization, the utility of additives, and green solvents. And although many efforts have been made to improve the properties of bio-based polymeric membranes, the menu of applications is still limited, and the possibility of scaling up, more research is needed, including circular bio-economy concepts.

## Figures and Tables

**Table 5:** Environmental application of bio-based membranes.

Application	Mechanism	Bio-Based Polymer	Contaminants	Removal Efficiency (%)	References
Water treatment	Recovery	Cellulose acetate modified by sulfonic acid functionalized dendrimer-grafter cellulose	Pb(II)	>98	[141]
			Na_2_SO_4_	97	
			Rose Bengal dye	98.6	
			Reactive Blue 50 dye	96.8	
			Azithromycin	88.6	
			BSA	99	
		Cellulose acetate with Ti_2_AIN MAX	Reactive black 5	70.7	[142]
			Reactive red 120	93.5	
			BSA	>98	
		PLA/PBS with cellulose	Cobalt ion	83	[143]
			Nickel ion	84	
Potable water purification	Adsorption	Chitosan-functionalized-PVA/Sodium alginate	As(III)	50–90	[144]
Wastewater treatment		Chitosan modified with 1-butyl-3-methylimidazolium acetate	Cr(VI)	87	[145]

BSA: bovine serum albumin. PVA: polyvinyl alcohol.

## Data Availability

Not applicable.
